# Decreased adiponectin and increased inflammation expression in epicardial adipose tissue in coronary artery disease

**DOI:** 10.1186/1475-2840-10-2

**Published:** 2011-01-12

**Authors:** Yuan Zhou, Yutao Wei, Lei Wang, Xianguo Wang, Xinling Du, Zongquan Sun, Nianguo Dong, Xinzhong Chen

**Affiliations:** 1 Department of Cardiovascular Surgery, Union Hospital, Tongji Medical College, Huazhong University of Science and Technology, Wuhan, 430022, Hubei, China; 2 Department of Thoracic and Cardiovascular Surgery, First Hospital Affiliated to Medical College of Shihezi University, Shihezi, 832008, Xinjiang, China; 3 The First College of Clinical Medical Science, China Three Gorges University, Yichang, 443003, Hubei, China

## Abstract

**Background:**

Disorders of endocrine substances in epicardial adipose tissue are known causes of coronary artery disease (CAD). Adiponectin is associated with cardiovascular disease. However, expression of adiponectin in epicardial adipose tissue and its function in CAD pathogenesis is unclear. This study investigates adiponectin expression in epicardial adipose tissue in CAD patients.

**Methods:**

Vessels or adipose tissue samples collected from CAD patients and non-CAD controls were examined after immunochemical staining. Adiponectin, cytokines of interleukin-6 (IL-6) and necrosis factor-α (TNF-α) and toll-like receptor 4 (TLR4) expression level in adipose tissue were measured using real-time quantitative RT-PCR. Adiponectin concentrations in peripheral and coronary sinus vein plasma were measured with enzyme-linked immunosorbent assay. Peripheral vein plasma biochemistries were performed with routine laboratory techniques. Monocytes were collected from blood using lymphocyte separation medium. Expression level of cytokines and transcription factor NF-κB were measured to learn the effect of adiponectin on stearic acid-stimulated monocytes. Percentage of TLR4 positive monocytes was analyzed using flow cytometry.

**Results:**

Histological examination revealed increased macrophage infiltration into epicardial adipose tissue of CAD patients. Decreased adiponectin displayed by real-time quantitative RT-PCR was associated with enhanced cytokines of IL-6 and TNF-α or TLR4 expression level in epicardial adipose tissue, suggesting decreased circulating adiponectin may be useful as a more sensitive predictor for coronary atherosclerosis than routine laboratory examinations. Adiponectin suppressed secretion of IL-6 and TNF-α in stimulated monocytes and TLR4 was expressed on cell surfaces.

**Conclusions:**

Endocrine disorders in epicardial adipose tissue are strongly linked to CAD, and adiponectin has a protective effect by inhibiting macrophage-mediated inflammation.

## Background

Obesity plays a causative role in the pathogenesis of coronary artery disease (CAD), including primary atherosclerotic and restenotic changes after coronary artery bypass grafts (CABG). Adipose tissue is an organ that stores fat, as well as an active endocrine organ that secretes various types of bioactive molecules, including leptin [1], adiponectin [2], resistin [3], plasminogen activator inhibitor-1 [4,5], apelin [6], Tumor necrosis factor-α (TNF-α) [7] and interleukin-6 (IL-6) [8]. Fat distribution also influences the occurrence and development of cardiovascular disease [9-11]. Compared to people with fat localized to the buttocks and hips, those with central obesity (i.e., with a larger waist circumference) are at higher risk, suggesting that visceral fat has different biological characteristics and plays a more important role in pathopoiesis compared with subcutaneous fat.

Epicardial adipose tissue has properties of visceral fat [9]. Recent research has shown that disorders of endocrine substances secreted by this tissue can induce inflammation that contributes to the occurrence of cardiovascular disease [12]. However, its function in the progression of atherosclerosis and the potential endocrine function of myocardial metabolism are still under observation, and the molecular mechanism by which adipocyte factor acts on myocardial tissue and the coronary artery has not be confirmed.

Adiponectin is one of several important active cytokines secreted from adipose tissue. Epidemiologic studies have associated low-circulating levels of this adipokine with various cardiovascular diseases including CAD [13]. However, to our knowledge, adiponectin secretion in epicardial adipose tissue has not been reported. High concentrations of free fatty acid (FFA) can stimulate the conversion of monocytetomacrophage and promote transformation of macrophages into foam cells in an inflammatory microenvironment, which contributes to the formation of coronary atherosclerosis [5]. From this point of view, we have investigated adiponectin expression in epicardial adipose tissue and the effect of adiponectin on FFA-stimulated monocytes. Moreover, we have analyzed the changing of toll-like receptor 4 (TLR4) on the surface of monocytes to explore the potential mechanism underlying the effect of adiponectin.

## Materials and methods

Patients signed a written consent conform for tissue collection according to a protocol approved by the Ethic committee of Human Investigation of Union Hospital, Huazhong University of Science and Technology (HUST), which conforms to the Helsinki Declaration. Donation procedures complied with the laws of the P.R. China, and the specimens obtained were registered with the relevant governmental authorities of Hubei Province.

### Procurement of specimens

Eleven coronary artery samples (left anterior descending artery, LAD) combined with the surrounding adipose tissue were obtained from human remains voluntarily supplied by the Department of Anatomy, Tongji Medical College, HUST. Twelve internal mammary arteries, six radial arteries and 14 greater saphenous veins were obtained as bridge vessels from patients who were scheduled to undergo CABG surgery, before starting cardiopulmonary bypass (CPB) surgery. All collected vessels were fixed and embedded in paraffin for sectioning and immunochemical staining. Thirty-four patients who had been planning cardiac surgery first received selective coronary angiography (CAG). They were divided into a CAD group (n = 23) and a non-CAD control group according to the results of the CAG. Biopsy samples of epicardial adipose tissue (average 0.5-1.0 g) from all subjects and of subcutaneous fat from around the greater saphenous vein of CAD subjects (who planned to use this vein as a bridge vessel) were taken prior to heparin administration for CPB surgery. Each single tissue sample from one subject was divided into two parts, one part was fixed and embedded in paraffin for sectioning and immunochemical staining, and the other was shock-frozen and immediately stored in liquid nitrogen for total RNA extraction. Pectoral subcutaneous adipose tissues were also obtained from four patients without heart diseases with body mass indexes (BMI) ranging from 19 to 23 kg/m^2 ^who were undergoing non-cardiac surgery, and these tissue samples were cryopreserved in profound hypothermia as described above.

### Histological and immunohistochemical examination

The paraffin-embedded sections of vessels were stained with hematoxylin and eosin (H&E). The eleven coronary artery samples were then divided into CAD group and control group according to the results of pathological examination. For all vessels and adipose tissue samples, 5-μm-thick whole paraffin-embedded cut sections on polylysine-coated glass slides were incubated overnight at 4°C with phosphate buffered saline (PBS) containing 0.5% bovine serum albumin (Sigma-Aldrich, St. Louis, MO, USA) (PBS/BSA) to block nonspecific binding. The specimens were then incubated with rat polyclonal anti-CD68 antibody (Abcam, Cambridge, MA, USA; 1:50 in PBS/BSA) overnight at 4°C in a moist incubation chamber. After washing three times in PBS, specimens were incubated with the secondary antibody against the rat polyclonal antibody produced in goat (Boster, Wuhan, China; 1:500 in PBS/BSA). The slides were again washed in PBS three times and incubated with SABC complex diluted 1:500 for 30 min at room temperature. After a new wash in PBS, the reaction was developed with chromogen solution consisting of 0.03% hydrogen peroxide. Color intensity was monitored under a light microscope and compared with positive controls included in each reaction. The specimens were then washed under running water for 10 min, counterstained with Harris hematoxylin for 20 seconds, again washed under running water, dehydrated in ethanol, cleared in xylene, and mounted in Permount resin. The immunostained sections were analyzed under Olympic MX-50 optical microscope. Positive immunostaining for the specific antibody was analyzed quantitatively in five fields of the lesion area at large magnification (4 × 100) using a graded grid divided into 10 × 10 subdivisions and comprising an area of 0.0625 mm^2^.

### Real time quantitative RT-PCR analysis

Total RNA from frozen adipose tissues stored in liquid nitrogen was extracted using TRIzol reagent (Invitrogen, CA, USA) following the manufacturer's instructions. The reverse transcription reactions were conducted with Transcriptor First Strand cDNA Synthesis Kit (Roche, Indianapolis, IN, USA). The oligonucleotide primer sequences were designed by Premier Primer 5.0 software as Table 1. β-actin was used as an internal control. The synthesized first-strand cDNA samples were subjected to real-time PCR with SYBR Green PCR Master Mix (Toyobo Bio-Technology, Shanghai, China) and PCR was performed using ABI Prism 7700 Sequence Detector (Applied Biosystems, Tokyo, Japan). The integrity of PCR products was confirmed by dissociation curve analysis using 2.0 software (Applied Biosystems). The threshold cycle (C_T_) values were determined and relative gene expression was calculated using the formula 2^-ΔΔCT ^with pectoral subcutaneous adipose tissues obtained from non-CAD patients during non-cardiac surgery.

**Table 1:** Real-time quantitative RT-PCR Primers

Gene title	Accession number	Primer	Amplicon size (bp)
Adiponectin	XM_290602	Forward 5'-GTCCTAAGGGAGACATGG-3'	302
		Reverse 5'-GATCTTCATGAGGTAGTCAGT-3'	
IL-6^a^	NM_000600	Forward 5'-GTGAAAGCAGCAAAGAGGCA-3'	276
		Reverse 5'-TTGGGTCAGGGGTGGTTATT-3'	
TNF-α^b^	NM_000594	Forward 5'-CCGAGTCTGGGCAGGTCTA-3'	201
		Reverse 5'-CGAAGTGGTGGTCTTGTTGC-3'	
TLR4^c^	NM_138554	Forward 5'-GGCTCACAATCTTATCCAATCT-3'	129
		Reverse 5'-TGATGTAGAACCCGCAAGTC-3'	
β-actin	NM_001101	Forward 5'- CCAACCGCFAFAAFATGACC-3'	175
		Reverse 5'-GATCTTCATGAGGTAGTCAGT-3'	

a Interleukin-6

b Tumor necrosis factor-α

c Toll like receptor 4

### Laboratory methods and enzyme-linked immunosorbent assay

Peripheral vein blood was drawn in the fasting state, and serum biochemistries were performed with routine laboratory techniques for fasting plasma glucose (FPG), triglycerides (TG), total cholesterol (TC), low-density lipoprotein cholesterol (LDL-C), high-density lipoprotein cholesterol (HDL-C), apolipoprotein AI (apo AI), apolipoprotein B (apo B) and lipoprotein (a) (Lp(a)). Blood samples for plasma adiponectin measurement from coronary sinus were collected during right heart catheterization. Adiponectin concentrations in peripheral and coronary sinus vein plasma were determined by enzyme-linked immunosorbent assay (ELISA) according to the protocol provided by the manufacturer (ALPCO Diagnostics).

### Preparation of human monocytes

Twenty milliliters of whole blood donated by healthy volunteers was randomly obtained from the Wuhan Blood Centre, which complied with the Blood Donation Law of the P.R. China. Monocytes were separated from anticoagulated whole blood using human lymphocyte separation medium according to the manufacturer's instructions (PAN Biotech, Aidenbach, Germany). Before treatment, the separated cells were suspended in a culture bottle with high-glucose RPMI1640 medium (Gibco, Invitrogen, Australia) supplemented with 10% fetal bovine serum (Gibco) and maintained at 37°C in a humidified 5% CO_2 _atmosphere overnight. After disposal of non-adherent cells, and thoroughly washing the adherent cells with phosphate-buffered saline (PBS) twice, cells were recovered using 0.5% trypsin (Gibco). For evaluating the percentage of monocytes and the viability of collected cells, immunofluorescence and trypan blue staining were performed. The concentration of cells was then adjusted to 1 × 10^6^/ml to reserve.

### Experiment on stearic acid-stimulated monocytes

Isolated monocytes were equilibrated to a steady state at densities of 4,000 cells per well in 96-well culture plates for 4 hours, then the pro-inflammatory effects of stearic acid (SA) and anti-inflammatory effects of adiponection on monocytes were tested. First, monocytes were cultured in the presence of 100 ng/ml lipopolysaccharide (LPS) (Sigma-Aldrich, St. Louis, MO, USA) (positive control), SA (Sigma-Aldrich) at three different concentrations (0.1, 0.5 and 1.0 mM) or PBS (negative control) for 24 hours. Meanwhile, three different final concentrations of globular adiponectin (gAd) (5, 10 and 20 μg/ml) (Peprotech, Rocky Hill, NJ, USA) or PBS (negative control) were added as follows. After incubation for 6 hours, monocytes were stimulated by 0.5 mM SA for 24 hours. In the next experiment, cultured supernatants were collected and TNF-α and IL-6 concentrations were quantified according to the manufacturer's instructions (R&D Systems, Minneapolis, MN, USA). In these two treatments, the activity of the transcription factor NF-κB was also measured in monocytes using Histostain-Plus Kit, following manufacturer's instructions (Invitrogen, CA, USA).

### Flow cytometry analysis

Monocytes were plated into a 6-well culture plate at a density of 1 × 10^6^/well and equilibrated at 37°C for 2 hours. Three different concentrations of gAd (5, 10 and 20 μg/ml) or PBS (negative control) were added and stimulated by 0.5 mM SA as described above. The collected cells were then stained with a dual-color antibody panel composed of CD14 (eBioscience, San Diego, Calif) and TLR-4 (BD PharMingen, San Diego, CA, USA ) and analyzed by 6-fluorescent-parameter BD LSRII (BD Biosciences, Sparks, MD, USA).

### Statistical analysis

Data were presented as means ± SEM. When comparing groups, one-way ANOVA was used to test for significance. A value of p < 0.05 was considered statistically significant.

## Results

### Histological characteristics

**Figure 1:**
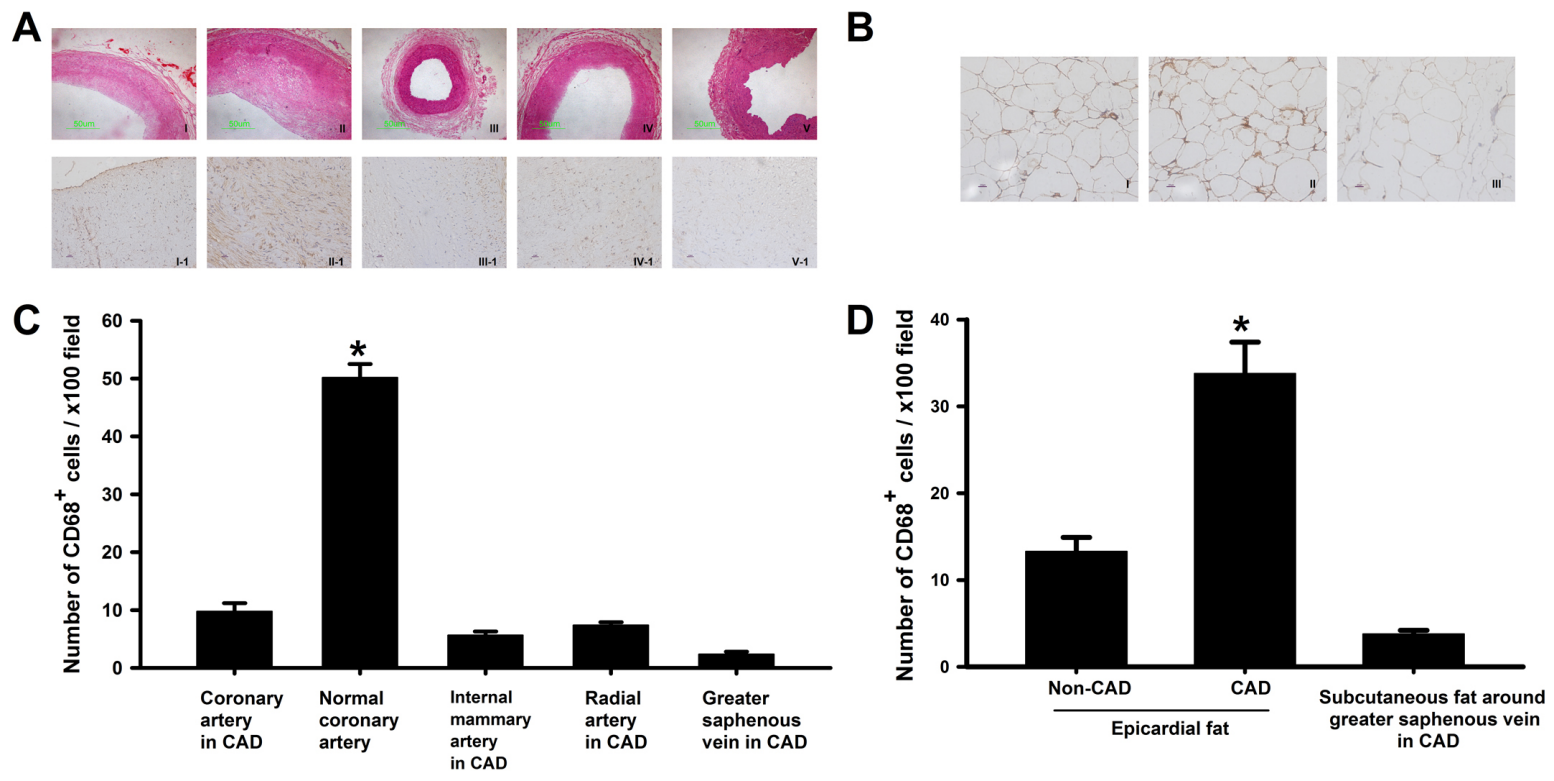
**Histological studies on blood vessels and adipose tissues**. **(A) I**: Normal coronary arteries presented intact endometrium and luminal patency; **II**: Atherosclerosis in the coronary artery, with a degree of stenosis of approximately 75%; **III-IV**: Internal mammary artery and radial artery from CAD patients, both showing intact endometrium and luminal patency, respectively; **V**: Greater saphenous vein from CAD patients presented luminal patency; **I-1**:Endothelial cells were intact, SCMs and fibers of intercellular layer are orderly arranged, and scarcely any CD68^+ ^cells were observed in the adventitia; **II-1**: Large amounts of CD68^+ ^cells infiltrated the adventitia in coronary atherosclerosis, which formed an accumulation zone; **III-1~Ⅴ-1**:Internal mammary artery, radial artery and greater saphenous vein were present, respectively, and obvious infiltration of CD68^+ ^cells was not observed in the adventitia of each group. **I-1~Ⅴ-1 **immunohistochemical staining corresponding to the tissue stained by H&E. **(B) **The quantity of CD68^+ ^cells infiltrated into epicardial adipose tissue in CAD was significantly increased. The bar charts were showing the number of CD68^+ ^cells per ×100 field in each section of blood vessels **(C) **and adipose tissues **(D)**, respectively. Date represented the analysis of minimum of 8 random sections per sample. Values are mean ± SEM. *P < 0.05 vs normal coronary artery in C and epicardial adipose tissue in non-CAD patients and subcutaneous fat around greater saphenous vein in CAD patients in D. Abbreviations: CAD, coronary artery disease; SMCs, smooth muscle cells.

CD68 is especially expressed by the mononuclear phagocytic system and highly expressed on the surface of macrophages [14]. As shown in Figure 1A, no changes in the vascular endothelium, elastic fibers or adventitia (including from greater saphenous vein) were observed in samples from either the internal mammary artery or radial artery. However, atherosclerosis, elastorrhexis, a large amount of CD68^+ ^cell infiltration in the adventitia, and an obvious zone of CD68^+ ^cell accumulation was observed at the interface between the adventitia and epicardial adipose tissue in samples from CAD patients. In the above five types of blood vessels, the highest density of CD68^+ ^cells in coronary arteries from CAD patients is shown in Figure 1C. Meanwhile, the magnitude of CD68^+ ^cell infiltration into epicardial adipose tissue was significantly higher than that observed in samples either with or without subcutaneous fat around the greater saphenous vein in patients with CAD (Figure 1B and 1D).

### Gene expression profile in different adipose tissues

**Figure 2:**
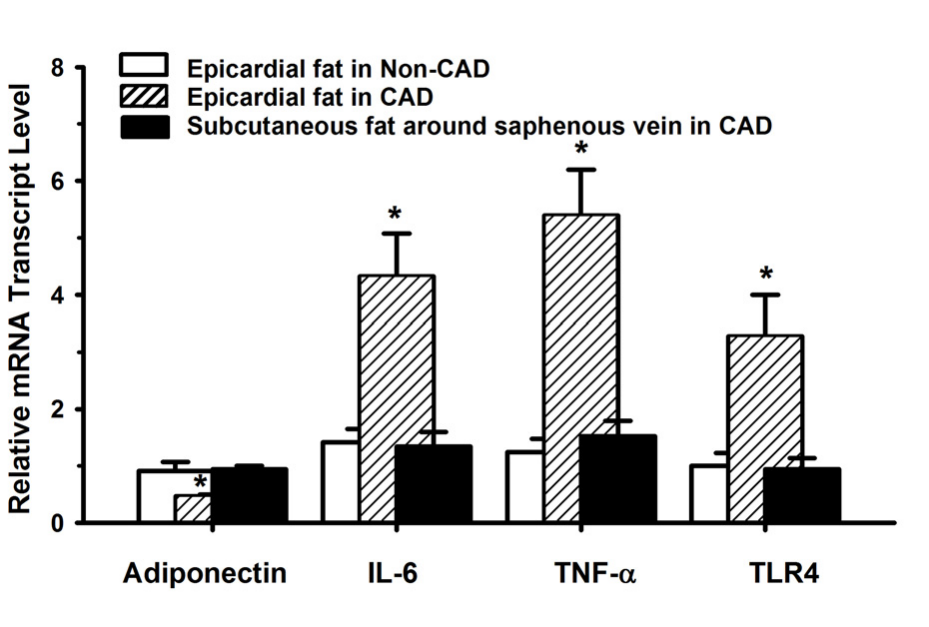
**Quantitative comparison of gene expression among adipose tissues**. After normalization to β-actin, relative adiponectin, IL-6, TNF-α and TLR4 mRNA transcription level were expressed as compared to pectoral subcutaneous adipose tissue obtained from four patients without heart diseases, which was set equal to 1. Relative adiponectin, IL-6, TNF-α and TLR4 mRNA expression was measured by real time quantitative RT-PCR in different adipose tissues. Results are the mean of triplicate measurements repeated twice. *P < 0.05 vs epicardial fat in non-CAD patients and subcutaneous fat around greater saphenous vein in CAD patients. Abbreviations: IL-6, interleukin-6; TNF-α, tumor necrosis factor-α; TLR-4, toll-like receptor 4; CAD, coronary artery disease.

By performing real-time quantitative RT-PCR, the expression levels of adiponetin, IL-6, TNF-α and TLR4 mRNA in different adipose tissues were examined. As shown in Figure 2, the expression patterns of these genes in epicardial adipose tissue in non-CAD patients were similar to those in subcutaneous fat around the greater saphenous vein in CAD patients. Comparing the two types of samples more closely, the gene levels in epicardial adipose tissue in CAD patients were rather different. Adiponectin mRNA was poorly expressed, but IL-6, TNF-α and TLR4 were highly expressed. It would seem that a strongly negative correlation would be expected between the levels of adiponectin mRNA and the other three genes.

### Decreased circulating adiponectin level is an independent risk factor for CAD

It has been reported that the plasma level of adiponectin in coronary circulation is significantly related to adiponectin expression in epicardial adipose tissue [15]. To investigate whether decreased adiponectin expression in epicardial fat can be used as a sensitive predictor of coronary atherosclerosis in non-obese patients, we examined the coronary sinus vein plasma levels of adiponectin and routine biochemical values, including TG, LDL-C, HDL-C, TC, FPG, apo AI, apo B and Lp(a), in peripheral vein of elderly male with body mass index (BMI) < 25.0, who received coronary angiography, a routine examination for patients over the age of 60 years undergoing cardiac surgery to exclude CAD. Subjects were divided into two groups. Within the CAD group, nine subjects were confirmed to have significant (>75%) narrowing of at least one major coronary artery; the non-CAD group included eleven subjects with smooth coronary arteries. As summarized in Table 2, there was no statistically significant difference between the two groups for TG, LDL-C, HDL-C, TC, FPG, apo AI, apo B and Lp(a), but the adiponectin concentrations in peripheral and coronary sinus vein plasma were both significantly lower in the CAD group suggesting that decreased circulating adiponectin may play an independent role in the pathogenesis of coronary atherosclerosis.

**Table 2:** Biochemical parameters in CAD and non-CAD patients

	CAD (n = 9)	non-CAD (n = 11)	P
age (years)	71 ± 9	69 ± 11	n.s.
BMI (Kg/m^2^)	23 ± 1.7	22 ± 2.1	n.s.
FPG (mM)	5.2 ± 1.4	5.1 ± 1.8	n.s.
TG (mM)	3.9 ± 1.6	4.0 ± 1.5	n.s.
TC (mM)	1.7 ± 0.6	1.6 ± 0.7	n.s.
HDL-C (mM)	1.1 ± 0.3	1.2 ± 0.5	n.s.
LDL-C (mM)	1.9 ± 0.7	2.0 ± 0.5	n.s.
apo AI (g/l)	0.9 ± 0.2	1.0 ± 0.3	n.s.
apo B (g/l)	0.8 ± 0.3	0.9 ± 0.1	n.s.
Lp(a) (mg/dl)	29 ± 17	32 ± 16	n.s.
peripheral plasma adiponectin (μg/ml)	5.9 ± 1.4	10.3 ± 2.2	< 0.01
coronary sinus plasma adiponectin (μg/ml)	4.5 ± 1.9	12.7 ± 3.1	< 0.01

Date are expressed as mean ± SD. Abbreviations: BMI, body mass index; FPG, fasting plasma glucose; TG, triglycerides; TC, total cholesterol; HDL-C, high-density lipoprotein cholesterol; LDL-C, low-density lipoprotein cholesterol; apo AI, apolipoprotein AI; apo B, apolipoprotein B; lipoprotein (a), Lp(a).

### Adiponectin suppresses inflammatory cytokines secretion and apoptosis of SA-stimulated monocytes

**Figure 3:**
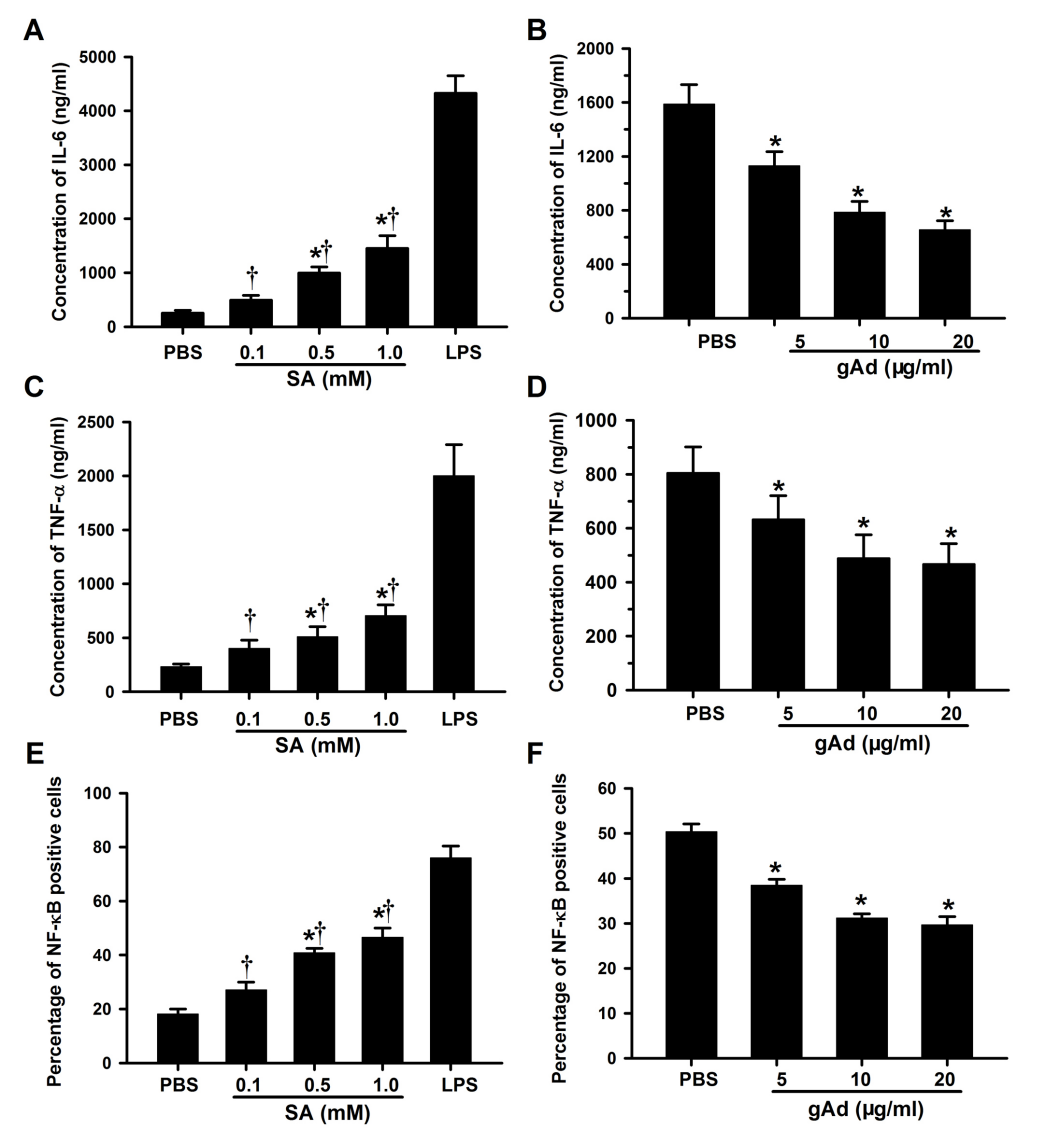
**Experiment on stimulated monocytes**. (A and C) Monocytes were treated with various concentrations of SA, and the histograms showed that obvious dose-effect relationships exist between TNF-α and IL-6 secretion by monocytes and SA; (B and D) Monocytes were treated with various concentrations of gAd along with 0.5 mM SA. The histograms showed that gAd had an inhibitory effect on monocyte secretion of TNF-α and IL-6. (E and F) SA could active NF-κB, and the process could be inhibited by gAd. PBS and LPS were used as negative and positive control, respectively. Data are mean ± SEM. *P < 0.05 vs negative control and ^†^P < 0.05 vs positive control. Triplicate experiments were performed with essentially identical results. Abbreviations: IL-6, interleukin-6; TNF-α, tumor necrosis factor-α; SA, stearic acid; gAd, globular adiponectin; PBS, phosphate buffered saline; LPS, lipopolysaccharide.

Using density gradient centrifugation techniques as described above, we successfully enriched monocytes from human whole blood. Studies on the expression of monoclonal antibodies demonstrate that CD14 is a specific marker for monocytic cells, so immunofluorescence was performed to identify the percentage of CD14 positive cells [16]. Results confirmed that the purity of monocytes in the isolated cells was more than 90% (data not shown) and that cell viability was over 95% as determined by staining with trypan blue. TNF-α and IL-6 are critical cytokines that respond to the progression of atherosclerosis [17]. To examine the proinflammatory effect of SA on monocyte secretion of TNF-α and IL-6, we added SA into the medium to a final concentration of 0.1, 0.5 and 1.0 mM. PBS and 100 ng/ml LPS were added as negative and positive controls, respectively. Compared with negative controls, SA had a stimulatory effect on monocyte secretion of TNF-α and IL-6, and an obvious dose-response relationships was observed between SA and both TNF-α and IL-6 (Figure 3A and 3C). Adioponectin is present in trimer form, and gAd is its proteolytic cleavage product [18]. To address the antiinflammatory effects of adiponectin, different concentrations of gAd were tested. Compared with PBS, gAd can inhibit stimulated monocytes secreting TNF-α and IL-6 (Figure 3B and 3D). Consistent with these results, SA can active NF-κB and induce monocyte apoptosis, which can be inhibited by gAd (Figure 3E and 3F).

### Adiponectin suppresses TLR4 expression of SA-stimulated monocytes

**Figure 4:**
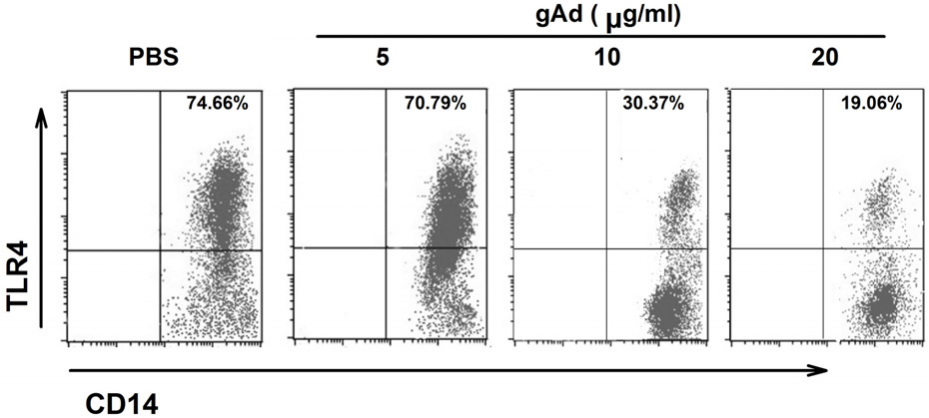
**Two-color flow cytometry analysis of SA-stimulated monocytes treated with different concentrations of gAd**. This figure shows results of immunoblots analyzed in SA-stimulated monocytes treated with gAd. Representative scatter plot of flow cytometry analysis for CD14 and TLR4 expression in treated monocytes and only CD14 positive cells are displayed. The percentage of expression of TLR4 in CD14 positive cells decreased as the concentration of gAd increased. Abbreviations: SA, stearic acid; gAd, globular adiponectin; TLR4, toll-like receptor 4.

As analyzed by real time quantitative RT-PCR, epicardial adipose tissue exhibited a relatively high level of TLR4 mRNA consistent with inflammatory cytokines IL-6 and TNF-α. However, the level of adiponectin mRNA is relatively low. To understand the relationship between expression levels of TLR4 mRNA and the inflammatory cytokines, we used CD14 and TLR4 antibodies applicable for flow cytometry (FCM). FCM of monocytes was observed on samples collected from three different gAd concentration groups after stimulation with SA. We observed a significant decrease in the ratio of TLR4 positive cells as the treatment concentration of gAd increased from low to high (Figure 4). These results strongly suggest that adiponectin down-regulates the expression of TLR4 when monocytes are treated with SA.

## Discussion

The present study reports the results of investigating adiponectin and inflammatory cytokines secretion in epicardial adipose tissue in non-CAD and CAD patients. A low level of adiponectin was found in the latter, coupled with a high level of IL-6 and TNF-α. Consistent with our findings, Baker *et al. *reported that expression of other cytokines in epicardial adipose tissue, including resistin, CD45 and angiotensinogen, were also significantly different in CAD patients compared to non-CAD controls [19]. These results suggest that change in the excretion profile of cytokines is a key player in the progression of CAD.

With the use of CD68 as a macrophage marker, we found that the level of macrophage infiltration in epicardial adipose tissue in CAD patients is higher than in non-CAD patients. *In vitro *and *in vivo *evidence has been provided to show that FFA, which is released from the adipocytes contributing to macrophage-induced adipocyte lipolysis, may play a role in the infiltration of macrophages into adipose tissue [20,21]. Macrophages participate in an arterial immune-inflammatory reaction triggered by LDL-C [22]. Data in this study demonstrate that the expression of IL-6 and TNF-α increases in epicardial adipose tissue in CAD patients resulted in aggregation of abundant macrophages, suggesting that IL-6 and TNF-α take part in this process.

Adiponectin is an important secreted serum protein [12,23,24]. The human adiponectin gene is located on chromosome 3q27 [25], and it codes for a 244 amino acid polypeptide with a signal sequence [23]. It is present in human plasma and adipose tissue and is induced during adipogenesis [12]. Adiponectin has beneficial roles for the vascular system and it has been proven that decreased plasma levels of adiponectin are associated with vascular events such as diabetic foot [26] and coronary atherosclerosis [27]. The current study presents data from epicardial adipose tissue, which is metabolically active and generates various bioactive molecules [9]. Results of this study demonstrated a significant inverse relationship between the level of adiponectin and IL-6 or TNF-α.

We also focused on the role of reduced expression of adiponectin in epicardial adipose tissue in the pathogenesis of coronary atherosclerosis, although previous study has proven that abdominal adiposity might play a more significant role [28]. We found that decreased circulating adiponectin-induced coronary atherosclerosis might be independent of plasma levels of glucose and lipids, which suggests that high glucose and lipids concentrations are not necessary for disorders of epicardial adipose tissue biology or CAD. Otherwise, our data strongly suggest that epicardial fat is an important secretion source of adiponectin and that the peripheral plasma adiponectin level at least partly influenced by it. Adiponectin levels in the peripheral circulation were also better related to intracoronary adiponectin [15,29]. Given a recent report [30], we conclude that a combination of the concentration of peripheral plasma adiponectin and the thickness of epicardial adipose tissue may be used as a highly sensitive predictor for coronary atherosclerosis in non-obese populations.

*In vitro* cell experiments showed that adiponectin could suppresses the cytokines secretion of stimulated monocytes and that the concentration of endogenous ligand was essential for activation of the TLR4-mediated signaling pathway. Plasma SA was elevated more than 2 to 3 times in samples from obese patients compared to normal samples [31]. An excessive level of SA in plasma can contribute to expression of TLR4 and activation of the TLR4-mediated signaling pathway, which leads to macrophage migration and subsequent triggering of AS [32]. Our study shows that adiponectin can down-regulate TLR-4 expression, which decreases inflammation by reducing SA combined with TLR4.

Some limitations of this study must be considered. Although we revealed that there was no obvious change in the biological properties of subcutaneous fat, endocrine disorders in other visceral fat such as abdominal fat could not be excluded in non-obese CAD patients, giving that Cheng KH *et al. *demonstrated that the tissue level of adiponectin in abdominal fat was significantly lower than epicardial fat in CAD patients [28]. Thus, further studies are needed to characterize the relative contribution of epicardial fat. In addition, Almeda-Valdes P *et al. *showed that total adiponectin, high molecular weight adiponectin (HMWA) and the HMWA/total adiponectin index had similar utility for identifying insulin resistance and metabolic disturbances [33], but whether clinical differences exist between them for predicting risk of coronary atherosclerosis is still unclear.

## Conclusions

In conclusion, this research confirms that immunologic endocrine disorders in epicardial adipose tissue are strongly linked to CAD. High production of proinflammatory factors in CAD patients is due to changes in the biological properties of epicardial adipose tissue, which may be independently associated with increased plasma glucose and lipid levels. Otherwise, obesity-associated endocrine disorders in adipocytes can activate monocytes and subsequently trigger atherosclerosis, and adiponectin can inhibit this process through decreasing TLR4 expression on macrophage/monocytes. Concern is increasing about the role of epicardial adipose tissue in the development of CAD. A more thorough understanding of local myocardial inflammation and ischemia induced by epicardial adipose tissue will provide clues to the efficient prediction and treatment of CAD.

## Abbreviations

apo A1: apolipoprotein A; apo B: apolipoprotein B; CAD: coronary artery disease; CABG: coronary artery bypass grafts; FFA: free fatty acid; FPG: fasting plasma glucose; HDL-C: high-density lipoprotein cholesterol; gAd: globular adiponectin; IL-6: interleukin-6; LPS: lipopolysaccharide; Lp(a): lipoprotein (a); LDL-C: low-density lipoprotein cholesterol; PBS: phosphate buffered saline; SA: stearic acid; TNF-α: tumor necrosis factor-α; TC: total cholesterol; TG: triglycerides.

## Competing interests

The authors declare that they have no competing interests.

## Authors' contributions

YZ, YW, XW and XC participated in the design of the study and acquisition of data. YZ, LW and XC participated in the analysis and interpretation of data. YZ, YW and XC participated in manuscript preparation. XD, ZS and ND participated in review of the manuscript. All authors have read and approved the final manuscript.
